# Solvothermal synthesis of NiWO_4_ nanostructure and its application as a cathode material for asymmetric supercapacitors†

**DOI:** 10.1039/c8ra09128e

**Published:** 2018-12-13

**Authors:** Jinjun Tian, Yan Xue, Xinping Yu, Yuanchao Pei, Hucheng Zhang, Jianji Wang

**Affiliations:** Collaborative Innovation Centre of Henan Province for Green Manufacturing of Fine Chemicals, Key Laboratory of Green Chemical Media and Reactions, Ministry of Education, School of Chemistry and Chemical Engineering, Henan Normal University Xinxiang Henan 453007 China huchengzhang66@163.com jwang@htu.edu.cn; School of Biological and Chemical Engineering, Nanyang Institute of Technology Nanyang Henan 473004 China

## Abstract

This study proposes a facile solvothermal synthesis of nickel tungstate (NiWO_4_) nanowires for application as a novel cathode material for supercapacitors. The structure, morphology, surface area and pore distribution were characterized and their capacitive performances were investigated. The results showed that the NiWO_4_ nanowires synthesized in ethylene glycol solvent could offer a high specific capacitance of 1190 F g^−1^ at a current density of 0.5 A g^−1^ and a capacitance retaining ratio of 61.5% within 0.5–10 A g^−1^. When used as a cathodic electrode of an asymmetric supercapacitor (ASC), the NiWO_4_ nanowire based device can be cycled reversibly in a high-voltage region of 0–1.7 V with a high specific capacitance of 160 F g^−1^ at 0.5 A g^−1^, which therefore contributed to an energy density of 64.2 W h kg^−1^ at a power density of 425 W kg^−1^. Moreover, 92.8% of its initial specific capacitance can be maintained after 5000 consecutive cycles (5 A g^−1^). These excellent capacitive properties make NiWO_4_ a credible electrode material for high-performance supercapacitors.

## Abstract 2

This study proposes a facile solvothermal synthesis of nickel tungstate (NiWO_4_) nanowires for application as a novel cathode material for supercapacitors.

## Graphical abstract

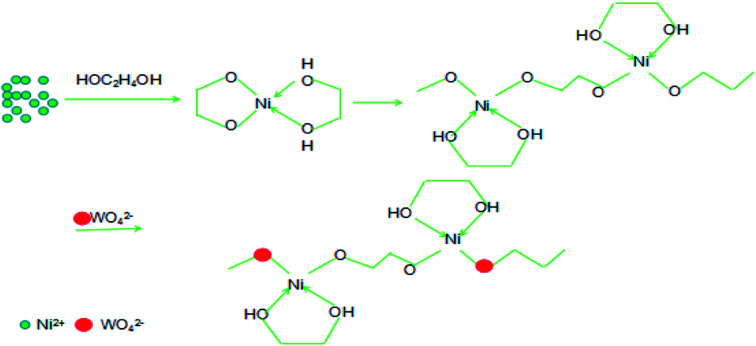

## 1. Introduction

With the ever-increasing energy consumption and the concomitant global warming and air pollution due to the over utilization of fossil fuels, clean and efficient energy storage devices have attracted unprecedented concern.^1,2^ Amongst the various energy devices, supercapacitors have received great attention by virtue of the high power density, charge–discharge rate, excellent cycling stability and environment friendliness.^3,4^ However, the major disadvantage of supercapacitors lies in their low energy density. So, more efforts have been devoted to the improvement of energy density without sacrificing their power density and cycle life.

Transition metal oxides/hydroxides such as NiO,^5^ Ni(OH)_2_,^6^ Co_3_O_4_ (ref. 7) are commonly employed faradic electrode material due to their low cost, low toxicity, and high specific capacity. Binary metal oxides NiCo_2_O_4_,^8^ ZnCo_2_O_4_,^9^ CdMn_2_O_4_ (ref. 10) and the related hydroxide electrode materials^11^ are reported to offer higher faradic capacitance relative to monometallic oxide as a consequence of the synergistic faradic contributions of both metallic elements. Metal tungstates (MWO_4_, where M = Co, Ni, Zn, Cu, Fe, Ca, Sr and Ba) represent a new class of semiconductor materials with excellent optical, electrical and sensor properties.^12,13^ Among these tungstates, NiWO_4_, CoWO_4_ and Bi_2_WO_6_ have gained considerable attention due to the excellent faradic activities and therefore energy storage capacities.^14–17^

The synthesis strategies of MWO_4_ mainly include the hydrothermal method,^16,18,19^ coprecipitation,^15^ solvothermal method,^12,20^ electrochemical deposition,^14,21^ microemulsion-mediated synthesis and other methods.^22^ By co-precipitation reaction, spherical NiWO_4_ composed of nanocrystals with average grain size about 10–40 nm were prepared.^23,24^ Using PEG or CTAB microemulsion as template, spherical NiWO_4_ architecture composing of primary nanocrystals were successfully formed.^25^ Assisted by polyethylene glycol (PEG) and triblock copolymer (pluronic 123) templates, wolframite structured NiWO_4_ nanocrystalline can be yielded.^26^ Templated by 3-(trimethoxysilyl) propyl hexadecyl dimethyl ammonium chloride (TPHAC), mesoporous nickel tungstate (*m*-NiWO_4_) with quasi-spherical morphology could be formed *via* hydrothermal reaction.^19^ Besides, octahedron NiWO_4_ nanocrystals with average crystallite size of 2.0 ± 0.1 μm can be facilely harvested by hydrothermal route.^27^

Different morphologies of NiWO_4_ can be prepared onto various substrates. Self-assembled NiWO_4_ nano-nests grown on a Ti substrate with perfect single-crystalline structure were synthesized by a hydrothermal process.^28^ Amorphous NiWO_4_ nanostructures with burl-like morphologies were successfully synthesized on a flexible conductive fabric (CF) substrate adhered seed-coated, which exhibited remarkable electrochemical properties with high specific capacitance (1190.2 F g^−1^ at 2 A g^−1^), excellent cyclic stability (92% at 10 A g^−1^), and good rate capability (765.7 F g^−1^ at 20 A g^−1^) in 1 M KOH electrolyte solution.^14^ Loose 3D network structure formed by the overlap of NiWO_4_ nano-particles and rGO sheet was successfully synthesized through solvothermal method, which provided a fast path for the transport of electrolyte ions, and thus facilitated the redox reaction.^20^ Carbon fibre was used to synthesized 3D structure of NiWO_4_/Ni/carbon fibre composite by combination of electrospinning with spray deposition and subsequent carbonization, which showed excellent capacity and cyclability.^29^

Although much progress has been made recently, the rational construction of NiWO_4_ with tailored architecture with high electrons/ions mobilities still remains to be the major research topic for sake of obtaining desired applicational performances. In this work, ethylene glycol was used to fabricate nanostructures by forming glycolate precursors because of its coordination interaction with transition metal ions. In absence of substrate, the NiWO_4_ nanowires were fabricated by a facile solvothermal reaction with ethylene glycol as solvent. The faradic electrode based on the as-prepared NiWO_4_ nanowires could offer high specific capacitance, excellent rate capability and good cycling performance.

## 2. Experimental

### 2.1 Materials

Na_2_WO_4_·2H_2_O (99%), NiCl_2_·6H_2_O (>97%, Macklin), ethylene glycol ((CH_2_OH)_2_), potassium hydroxide (KOH), polytetrafluoroethylene emulsion (PTFE, mass percent = 60%), activated carbon, nickel foam and all other reagents were purchased from Shanghai macklin Biochemical Co., Ltd. All reagents were used without further purification.

### 2.2 Synthesis of NiWO_4_ nanowires

Ethylene glycol was used solvent and ligand for preparation of NiWO_4_ nanowires. In brief, 2 mmol NiCl_2_·6H_2_O was dissolved into 35 ml ethylene glycol with vigorous stirring to form a transparent solution. Subsequently, 2 mmol Na_2_WO_4_·2H_2_O was added into form transparent reaction mixture. The obtained homogeneous solution was then transferred into a 50 mL Teflon-lined stainless steel autoclave and maintained at 180 °C. After being cooled to room temperature, the resulting product was rinsed repeatedly with deionized water and vacuumly dried overnight at 60 °C. The products prepared at different hydrothermal time were denoted as NiWO_4_-8, NiWO_4_-12, NiWO_4_-18, NiWO_4_-24, respectively, of which the suffix represent the hydrothermal duration, *e.g.* the NiWO_4_-8 stands for the product hydrothermally reacted for 8 h.

### 2.3 Characterizations

The chemical structure of composites were observed by X-ray diffraction (XRD, X'Pert PRO MPD, Cu Kα l = 1.54 Å) and X-ray photoelectron spectroscopy (XPS, Kratos Amicus X-ray photoelectron spectrometer with Mg Ka radiation under 2 × 10^−6^ Pa). The samples were measured from 10 to 80° (2*θ*) with steps of 4° min^−1^. The morphologies characteristics of composites were analyzed by field emission scanning electron microscopy (SEM), using a FEI Quanta 600 FEG system operated at 20 kV and transmitting electron microscopy (TEM), using a FEI Tecnai TF20 electron microscope by placing drops of the aqueous suspension onto copper grids. Nitrogen adsorption and desorption experiments were performed at 77 K on a SSA2000 system.

### 2.4 Electrochemical measurements

The electrochemical properties of the composites were evaluated by cyclic voltammetry (CV), galvanostatic charge–discharge (GCD) tests, and electrochemical impedance spectroscopy (EIS) on an electrochemical workstation (CHI660E, Chenhua). The working electrode was fabricated by mixing the active material, carbon black, and PTFE with a mass ratio of 75 : 15 : 10, which were coated onto a Ni foam (1 cm × 1 cm × 0.2 cm). The electrode was drying at 60 °C for 12 h. The loading mass of active material was ∼2 mg cm^−2^. Hg/HgO electrode, platinum plate (1 cm × 1 cm) and active materials were served as the reference electrode, counter electrode and working electrode, respectively. The electrochemical tests were performed in an aqueous electrolyte of 6 M KOH, within a potential window from 0 to 0.5 V. CVs were recorded in potential scan rate range of 5–100 mV s^−1^. EISs were recorded over 10^5^ to 0.01 Hz with an amplitude of the applied potential of 5 mV at the open circuit potential. The average specific capacitance (*C*_s_) was estimated from the discharge slope according to the following equation: *C*_s_ = *I*Δ*t*/*m*Δ*V*, where *I* is the discharge current (A), Δ*t* the discharge time (s), *m* the mass of active material on work electrode (g) and Δ*V*(V) the discharge voltage rage. The specific capacitance of the ASC (*C*_cell_), energy and power density were calculated respectively according to the following equations:

1 *C*_cell_ = *I*Δ*t*/*m*′*V*

2 *E* = *C*_cell_*V*/7.2

3 *P* = 3600*E*/Δ*t*

where *m*′ (g) is the total mess of electroactive materials in the positive and negative electrode, *V* (V) is potential window, *E* (W h kg^−1^) is the energy density, *P* (W kg^−1^) is the average power density.

## 3. Results and discussion

### 3.1 Characterizations of NiWO_4_ nanostructure

**Fig. 1:**
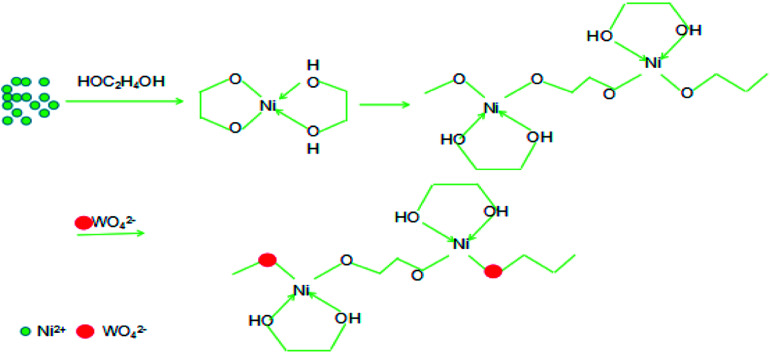
Schematic illustration of the formation of NiWO_4_ nanowires.

NiWO_4_ nanowires were prepared through a facile equimolar reaction between Na_2_WO_4_·2H_2_O and NiCl_2_·6H_2_O by solvothermal method free of any further annealing. Using ethylene glycol as solvent and ligand, glycolate precursors of Ni was formed through the coordination interaction, which is imagined to govern the release of Ni source and manipulate the final morphology of the resulted NiWO_4_ product. Fig. 1 outlines the plausible mechanism for the formation of NiWO_4_ nanowires. Ethylene glycol is a dihydric alcohol, the two hydroxyl groups can serve as chelation ligands to form O-heterocyclic Ni complex through Ni–O coordination bonds when Ni salt was dissolved in ethylene glycol. Undergoes solverthermal reaction at elevated temperature, the partial dissociation of Ni–O bond and the interlinking of adjacent complex units lead to the linear configuration of the Ni-glycolate coordination polymer precursors. Given the fact that hydroxyl group is not the most stable ligand for Ni^2+^ cation, the incorporated WO_4_^2−^ anions can react with the central Ni^2+^ cation by replacing of partial hydroxyl ligands through competitive reaction, thus primary NiWO_4_ nucleus were formed onto the coordination polymer chains. Undergoes the continued solvothermal process, the further aggregation, growth and the condensation of primary NiWO_4_ crystallites result in the linear morphology of the resultant NiWO_4_ product. In this solvothermally formation and growth procedure, the immature structure or the overcondensation may influence the final morphology and the surface utilization ratio of the NiWO_4_ product, the aging time is a significant parameter need to be well controlled. Considering the intrinsically high conductivity of NiWO_4_ (10^−7^ to 10^−3^ S cm^−1^ at different temperatures),^30^ the linear structure with efficient electrons migration channel, as well as the faradic activity of Ni element, high faradic capacitance can be ensured when employed as electrode material of supercapacitor.

**Fig. 2:**
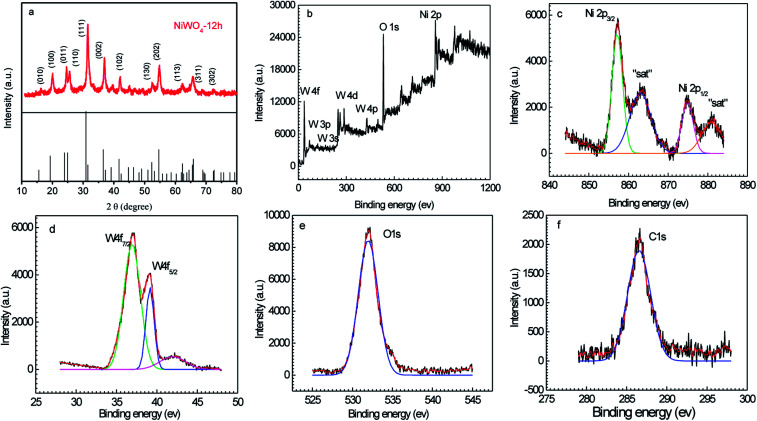
XRD patterns (a) and XPS spectra of NiWO_4_-12 sample (b–f).

Fig. 2a shows the XRD pattern of the typical NiWO_4_-12 sample, as seen, the XRD pattern comprises a series of diffraction peaks located at 15.6°, 19.27°, 23.96°, 24.9°, 30.9°, 36.5°, 41.6°, 52.3°, 54.6°, 62.3°, 65.8°, and 72.6°, which are corresponded respectively to the (010), (100), (011), (110), (111), (002), (102), (130), (202), (113), (311), and (302) crystallographic planes of NiWO_4_ (JCPDS# 15-0755).^14^ The surface bonding and element oxidation states of the NiWO_4_ samples were further characterized by XPS and the results were shown in Fig. 2b–f. As observed in Fig. 2b, the XPS survey scan spectrum indicates the presence of elemental Ni, W, O, and C according to their respective binding energies. The high-resolution Ni 2p, W 4f, and O 1s spectral curves were fitted well with the Gaussian curves as shown in Fig. 2c–e. From Fig. 2c, the Ni 2p spectrum mainly includes the spin orbit doublets of the Ni 2p_3/2_ and Ni 2p_1/2_ orbits at binding energies of 857.1 and 874.8 eV, respectively, as well as two shake-up satellite peaks, all of these features indicates the Ni elements mainly in form of Ni(ii) oxidation value.^31^ These results reveal that the Ni species are in the +2 oxidation state. Meanwhile, the high resolution XPS spectrum of W 4f showed spin–orbit splitting of W 4f_7/2_ at 37.0 eV and W 4f_5/2_ at 39.1 eV, implying that the W is in the +6 oxidation state in the prepared product. The O 1 s spectrum with a binding energy value of 532.0 eV was associated with the lattice O-bond with the W and Ni in NiWO_4_.^32,33^ To be mentioned, the weak C 1s peak is presumably derived from the incompletely rinced ethylene glycol molecules. The XPS, along with that of the XRD results evidence the successful formation of NiWO_4_ by solverthermal reaction.

**Fig. 3:**
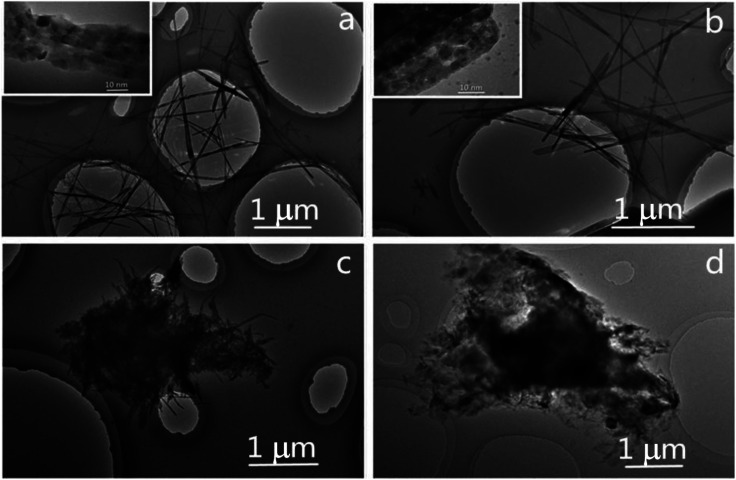
TEM of the NiWO_4_ samples prepared at different solvothermal time (a) NiWO_4_-8, (b) NiWO_4_-12, (c) NiWO_4_-18, (d) NiWO_4_-24.

Fig. 3 presents the TEM images of the NiWO_4_ samples synthesized for different solvothermal time. As depicted in Fig. 3a, the NiWO_4_-8 sample exhibits nanowires with an average diameter of 35 nm and average length of 3.3 μm, enlarged TEM image reveals that the nanowire is composed of stacked primary particles, which manifest the conversion and aggregation of NiWO_4_ particles from the linear Ni glycolate precursors. Undergoes prolonged solvothermal time course, the NiWO_4_-12 sample (Fig. 3b) exhibits the similar morphology, but the seemingly enlarged size of the primary particles and the enlarged inter-particle void suggest the more sufficient crystallization of the primary particles, the enlarged voids allows the more easily accessible of electrolyte and enhanced surface utilization ratio, which is especially beneficial for surface related applications. In the cased of NiWO_4_-18 prepared at more longer solvothermal reaction time (Fig. 3c), the sample displays irregularly intertwined nanowires with shorter length, which indicated the excessive solvothermal reaction causes the unreasonable condensation of the nanowires. The further prolonging of reaction time causes the further aging and the destroying of the nanowires, as shown in Fig. 3d, the NiWO_4_-24 mainly exhibits stacked short nanowires and particles with more ill-defined morphology, which manifest the excess solvothermal time is really disadvantageous for the nanowire morphology. Therefore, it could be concluded that the size and morphology of the precursor was significantly changed over reaction time. The morphological evolution of NiWO_4_ samples evidence the procedure in schematic illustration in Fig. 1, namely, the nanocrystal nucleies initially formed along the linear precursor, the subsequent aging caused the stacking and growing into nanowires, the overcondensation deteriorated the NiWO_4_ nanowires morphology.

**Fig. 4:**
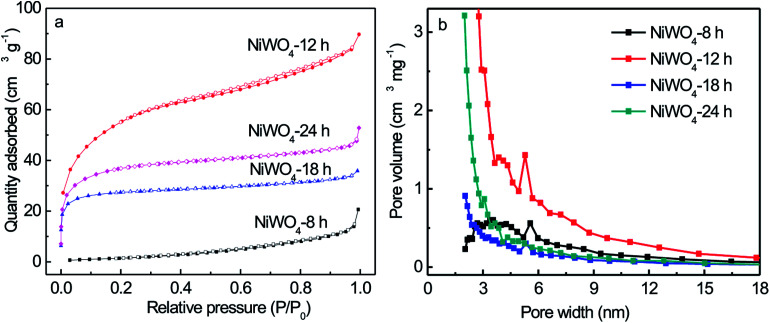
N_2_ adsorption–desorption isotherm (a), and pore size distribution (b) of NiWO_4_ samples prepared for different solvothermal time.

Furthermore, Brunauer–Emmett–Teller (BET) analyses were further performed to evaluate the surface area and pore size distribution of the NiWO_4_ samples. It could be observed in Fig. 4a, the isotherm of NiWO_4_-12 sample showed typical IV adsorption–desorption isotherms with a hysteresis in the range of 0.4–1.0 *P*/*P*_0_, indicating the presences of mesopores and macropores possibly formed by the loose stacking of constituent NiWO_4_ nanowires. The isotherm of NiWO_4_-8, NiWO_4_-18 and NiWO_4_-24 samples showed typical I adsorption–desorption isotherms, indicating mainly consists of microporous structure. The obtained NiWO_4_-12 sample was further confirmed by the pore size distribution at 3 to 7 nm and the calculated surface area was 199.7 m^2^ g^−1^. The BET specific surface area and the Barrett–Joyner–Halenda (BJH) desorption pore volumes of all NiWO_4_ samples were listed in Table S1.† The corresponding BJH pore size distribution curves revealed that the pore size distributions were non-uniform within the range of mesopores and macropores displayed in Fig. 4b.

### 3.2 Electrochemical performances of the NiWO_4_ samples

**Fig. 5:**
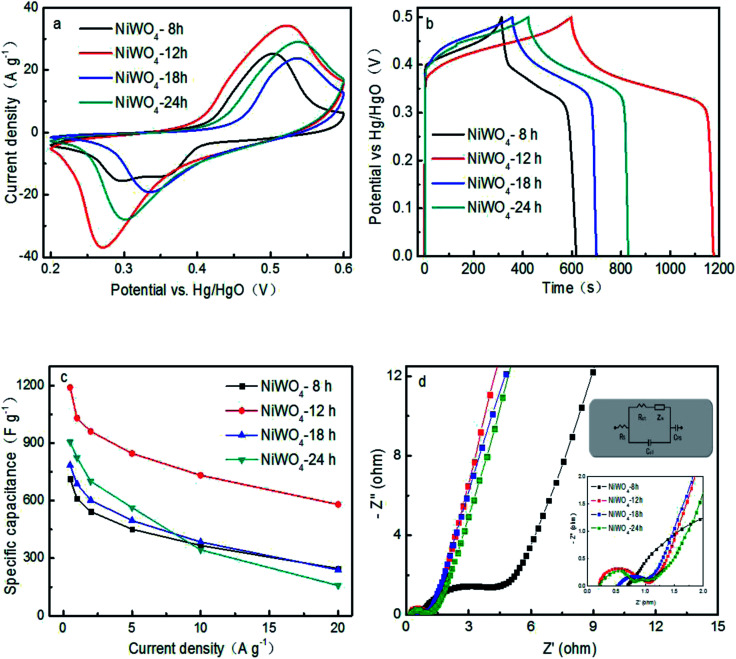
(a) CV curves at a scan rate of 10 mV s^−1^, (b) charging and discharging curves at 1 A g^−1^, (c) specific capacitance at different discharging currents density, and (d) EISs and inset for the equivalent circuit of NiWO_4_ samples prepared for different hydrothermal time in 6 mol L^−1^ KOH electrolyte.

In order to assess the feasibility of the fabricated NiWO_4_ in faradic electrode of supercapacitor, CV curves at a scan rate of 10 mV s^−1^ within the electrochemical window from 0.2 V to 0.6 V were carried out and the corresponding CVs were shown in Fig. 5a. Obviously, the shapes of CV curves were evidently different from that of EDLCs which were similar to an idea rectangular shape. All CV curves comprise a pair of well-defined redox peaks, suggesting that the measured capacitances were mainly governed by the faradaic redox mechanism. The current peaks were based on the reversible reactions of Ni^2+^ to Ni^3+^.^15^ Moreover, the integral area of NiWO_4_-12 is the largest, following with the NiWO_4_-24, NiWO_4_-18 and NiWO_4_-8. Fig. 5b exhibited the GCD curves of NiWO_4_ prepared for different hydrothermal time with as-triangular shape at 1 A g^−1^, due to the pseudocapacitance of reaction between Ni(ii) and Ni(iii), which was coincident with the CV curves. The specific capacitances of all samples calculated from the discharge curves at 1 A g^−1^ were presented in Fig. 5c. NiWO_4_-12 electrode exhibited the highest specific capacitance, which could be achieved 1190 F g^−1^ at a current density of 0.5 A g^−1^ and 1030, 960.8, 864, 732 and 580 F g^−1^ at current density of 1, 2, 5, 10 and 20 A g^−1^, respectively. Obviously, the specific capacitance of NiWO_4_ electrodes decreased with the increase of current density, which derived from the insufficient ion diffusion. The specific capacitance of NiWO_4_-24 was higher than that of NiWO_4_-18 and NiWO_4_-8.

EIS measurements were also carried out to evaluate the charge transfer and electrolyte diffusion in the electrode/electrolyte interface, as shown in Fig. 5d. Obviously, the EIS of NiWO_4_ electrode was composed of a semicircle at the high-frequency region and a straight line at the low-frequency region.^34^ It could be observed all plots reveal similar shape. The intercept at real axis (*Z*′) refers to the equivalent series resistance (*R*_s_), which mainly consists of the inherent resistance of the electrode materials, the electrolyte solution resistance, and the contact resistance at the interface.^35^ The *R*_s_ of NiWO_4_-12 (0.22 Ω) was lower than those of NiWO_4_-24 (0.27 Ω), NiWO_4_-18 (0.52 Ω) and NiWO_4_-8 (0.71 Ω), indicating that the low internal resistance and considerably outstanding conductivity.^36^ At the high frequency range, a small semicircle can be obviously observed, the diameter reflects the charge-transfer resistance (*R*_ct_) and derives from the double-layer capacitance and faradic reaction at electrode/electrolyte interface. Among all the samples, NiWO_4_-12 and NiWO_4_-24 exhibited the small values of *R*_ct_ (0.43 Ω and 0.46 Ω) at high frequency, implying the low charge transfer and diffusion resistance and fastest ion diffusion. Moreover, at the low frequencies, the slop of Nyquist plot curves of the NiWO_4_-12 was larger than that of NiWO_4_-8, NiWO_4_-18 and NiWO_4_-24, indicating its ideal capacitance performance. The high electrochemical performance, good conductivity of NiWO_4_-12 was attributed to minimize diffusion resistance to mass transport at the electrode/electrolyte interface for the smaller size using suitable solvents in solvothermal synthesis.

**Fig. 6:**
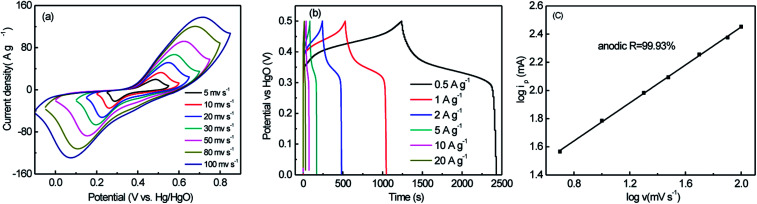
CV curves at different scan rate (a), charging and discharging curves at 1 A g^−1^ (b), and dependence of cathodic peak current on scan rate (c) of NiWO_4_-12 in 6 mol L^−1^ KOH electrolyte.

To further verify the pseudocapacitance contribution, typical CV curves of NiWO_4_ samples prepared using ethylene glycol as solvent at a variety of scan rates varying from 5 to 100 mV s^−1^ were shown in Fig. 6a. All CV curves exhibited a pair of well-defined redox peaks, signifying typical pseudocapacitive behavior. Moreover, the integral area CV curves of NiWO_4_ samples increased with the increase of the scan rates, and rapid current response on voltage reversal occured at each end potential, revealing its good electrochemical capacitance. Fig. 6b exhibited the GCD curves of NiWO_4_ with as-triangular shape at different current densities, due to the pseudocapacitance of reaction between Ni(ii) and Ni(iii), which was coincident with the CV curves. It was shown that the peak current (*i*_p_) in CVs increase with the increase of scan rate (*ν*), and followed a linear equation: log *i*_p_ = 0.67427 log *ν* +1.1022 (*R*^2^ = 0.9993, Fig. 6c). The linear slope closes to 0.5, indicated that the charge storage process is controlled by the bulk diffusion of electrolyte ions.

### 3.3 Electrochemical performances of the NiWO_4_//AC ASC

To obtain a high energy density, ASC was face-to-face assembled using NiWO_4_ nanowires and AC as cathode and anode electrodes with a cellulose paper sandwiched between them (Fig. 8). The assembly was immersed into 6 M KOH aqueous solution. The mass ratio of active materials on cathode electrode (m^+^) to anode electrode (m^−^) was set at 0.4 to match the charges according to the equation m^+^/m^−^ = C^−^/C^+^, where C^+^ and C^−^ respectively are the specific capacities of NiWO_4_ and activated carbon.

**Fig. 7:**
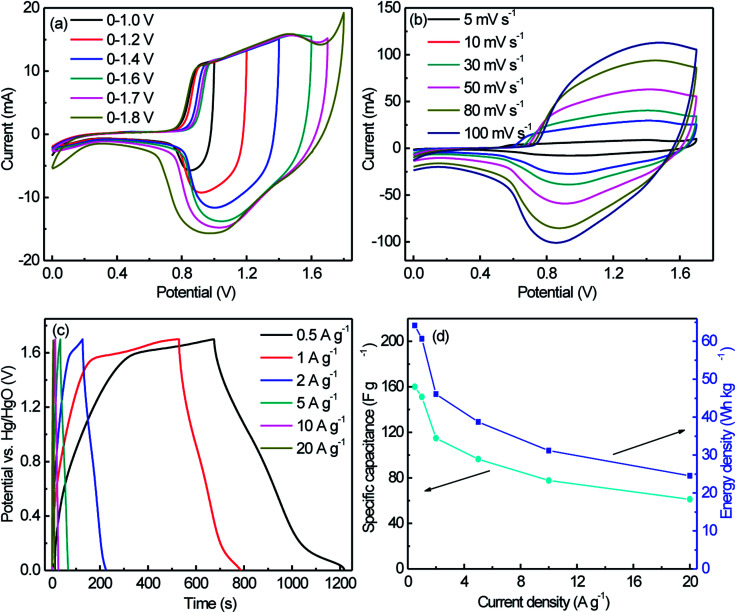
CV curves measured at 10 mV s^−1^ with different potential windows (a), CV curves measured at different scan rates (b), GCD curves measured at different current densities between 0 and 1.7 V (c), and the specific capacitance and energy density calculated from discharge curves at different current density (d) of NiWO_4_//AC ASC in 6 M KOH aqueous electrolyte.

**Fig. 8:**
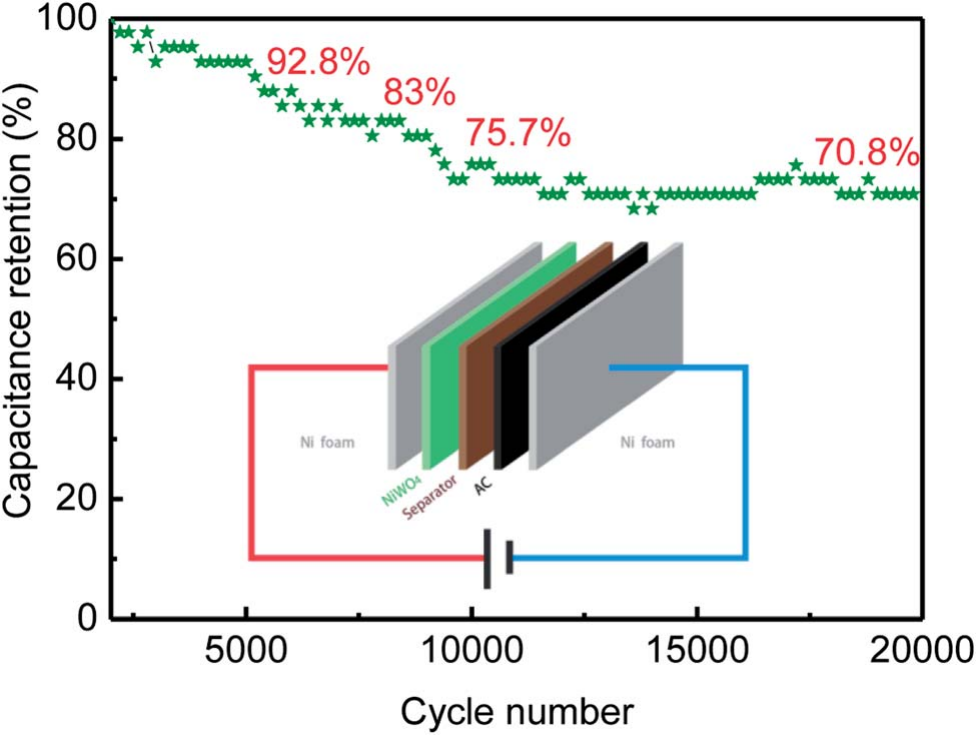
Cycle life of NiWO_4_//AC ASC in 6 M KOH aqueous electrolyte.

Fig. 7a gives CV curves of the NiWO_4_//AC ASC at different potential windows in 6 M KOH aqueous solution at 10 mV s^−1^. The assembled ASC showed a broad redox peak and could be cycled between 0 and 1.7 V with a good reversibility. Accordingly, the potential window of 0–1.7 V was chosen for the following investigation of the overall electrochemical performances of the ASC. Fig. 7b showed CV curves of the NiWO_4_//AC ASC at different scan rates in 6 M KOH aqueous solution. Fig. 7c showed the GCD curves of the NiWO_4_//AC ASC measured at different current densities in the 6 M KOH aqueous electrolyte, it could be observed that all the discharge curves were nearly symmetric with their corresponding charging counterparts, demonstrating the excellent electrochemical reversibility and good Coulombic efficiency. The specific capacitances calculated from the galvanostatic charge–discharge curves according to eqn (1) were 160, 151.2, 114.8, 96.5, 77.6 and 61.2 F g^−1^, corresponding to the current densities of 0.5, 1, 2, 5, 10 and 20 A g^−1^, respectively (Fig. 7d). It was clearly obtained that the capacitance decreased gradually with increase of current density, and 38.2% of capacitance was still retained when the current density increased from 0.5 to 20 A g^−1^, suggesting that the NiWO_4_//AC ASC possessed good rate capability. The energy density of the NiWO_4_//AC ASC had been shown in Fig. 7d, the *E*_cell_ achieved up to 64.2 W h kg^−1^ at *P*_cell_ of 450 W kg^−1^, and declined to 24.55 W h kg^−1^ with *P*_cell_ increases to 17 000 W kg^−1^, showing the high energy and power delivery abilities of the ASC and ranking a high level among the recently reported supercapacitors based on nanostructured NiWO_4_ (as the listed results shown in Table S2†).

The cycling stability of the asymmetric supercapacitor was further investigated by galvanostatic charge–discharge between 0 and 1.7 V at a current density of 5.0 A g^−1^. Results shown that the assembled supercapacitor retained 92.8% and 70.8% of initial capacitance even after 5000 and 20 000 consecutive cycles of charge–discharge testing, respectively, which displayed an excellent cycling ability of NiWO_4_//AC ASC. It is believed that the proper mass ratio of NiWO_4_ to AC can balance the charge storage in the positive and negative electrodes, and improve the electrochemical performances of the ASC. Based on the high energy and power delivering ability and the good cycleability, the NiWO_4_//AC ASC herein could serve as an efficient and long lifetime energy storage device.

## 4. Conclusions

In summary, the NiWO_4_ nanowires were successfully synthesized by a simple solvothermal method with ethylene glycol as solvent in absence of substrate. The NiWO_4_ nanowires were applied as electrode material for supercapacitors and exhibited a high specific capacitance of 1190 F g^−1^ at 0.5 A g^−1^ in a three-electrode system. The asymmetric supercapacitor device was successfully assembled using NiWO_4_ and AC as the cathode and anode, respectively. The device delivered a high energy density of 64.2 W h kg^−1^ at a power density of 425 W kg^−1^, and still maintained a power density of 17.0 kW kg^−1^ with an energy density of 24.55 W h kg^−1^. Furthermore, the supercapacitor also possessed an excellent long cycle life along with retained 92.8% and 70.8% of initial capacitance even after 5000 and 20 000 consecutive cycles in the high-voltage region of 0–1.7 V at 5 A g^−1^. These excellent properties make NiWO_4_ a promising electrode material for high-performance supercapacitor applications.

## Conflicts of interest

There are no conflicts to declare.

## Supplementary Material

RA-008-C8RA09128E-s001
