# Supplementary material for: Solvothermal synthesis of NiWO4 nanostructure and its application as a cathode material for asymmetric supercapacitors
Source: RSC Adv. 2018 Dec 13;8(73):41740–8. doi: 10.1039/c8ra09128e (PMC9092149; doi:10.1039/c8ra09128e)
Supplement: RA-008-C8RA09128E-s001 [file RA-008-C8RA09128E-s001.pdf]

Electronic Supplementary Material (ESI) for RSC Advances.
This journal is © The Royal Society of Chemistry 2018

Supporting Information (SI)

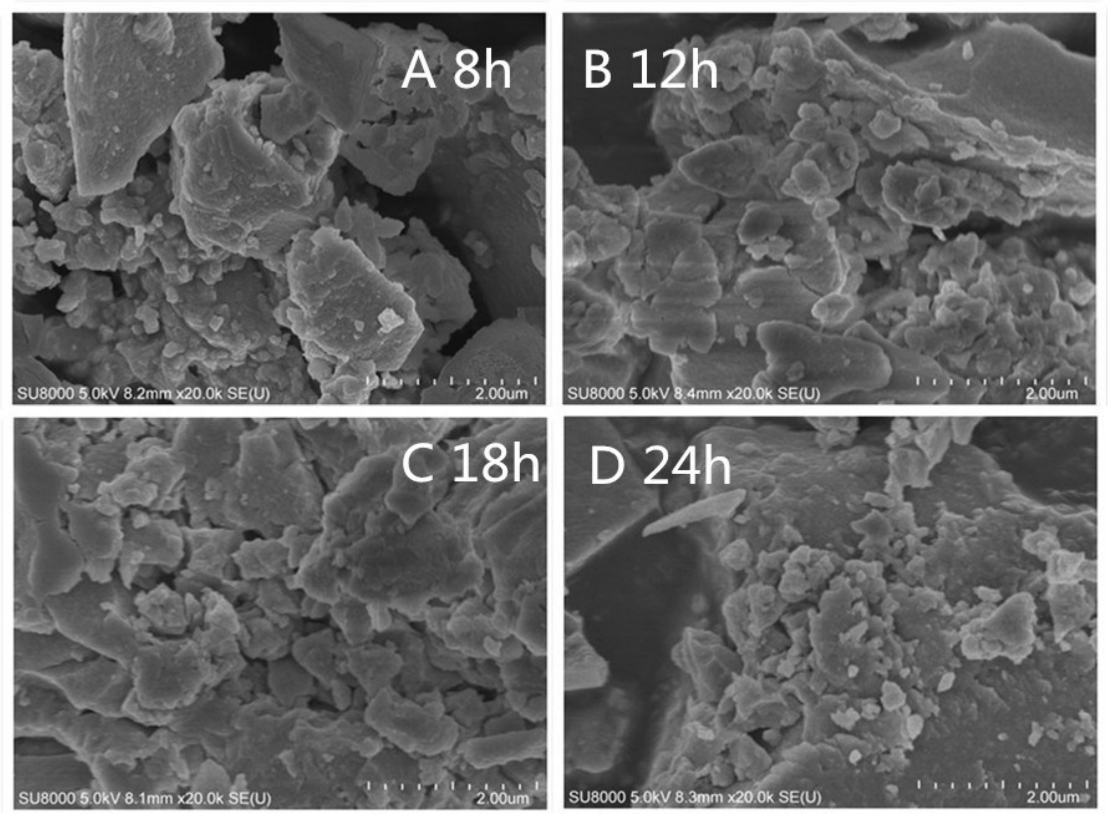

Figure S1. SEM image of (A) $NiWO_4$-8h, (B) $NiWO_4$-12h, (C) $NiWO_4$-18h and (D) $NiWO_4$-24h

Table S1 Structural paramenters of $NiWO_4$ different reaction time

Sample	$NiWO_4$-8h	$NiWO_4$-12h	$NiWO_4$-18h	$NiWO_4$-24h
Surface area/$m^2\ g^{-1}$	6.7	199.7	85.39	121.33
Volume/ml g^{-1}	0.032	0.1394	0.0557	0.0821

Table S2. Comparison of $NiWO_4$-based materials reported in our work and the recent $NiWO_4$ nanostructures from literatures.

Electrodes	Capacitance	Cycling number [Retention(%)]	References (Year)
**NiWO4/rGO**	1031.3 F g^{-1} at 0.5 A g^{-1}	5000, (99.24%)	[1] (2016)
**NiWO4@CoWO4**	196.7 C g^{-1} at 0.5 A g^{-1}	5000, higher than initial value	[2] (2016)
**NiWO4-CoWO4 //AC**	128.2C g^{-1} at 0.5 A g^{-1}	5000, (100%)	[2] (2016)
**NiWO4**	586.2 F g^{-1} at 0.5 A g^{-1}	1000, (90%)	[3] (2013)
**NiWO4 //AC**	71.1 F g^{-1} at 0.25 A g^{-1}	5000, (91.4%)	[3] (2013)
**NiCo2O4@NiWO4**	1384 F g^{-1} at 1 A g^{-1}□	6000, (87.6%)	[4] (2017)
**NiCo2O4@NiWO4 //AC**	131.7 F g^{-1} at 1 A g^{-1}	---	[4] (2017)
**NiWO4/Ni/CF**	792.2 F g^{-1} at 1 A g^{-1}	10,000, (174.8%)	[5] (2016)
**NiWO3 and NiWO4**	171.28 F g^{-1} at 0.25 A g^{-1}	---	[5](2016)
**NiWO4 /CF**	1,190.2 F g^{-1} at 2 A g^{-1}	1000, (92%)	[6] (2015)
**CoWO4@NiWO4**	746 F g^{-1} at 0.8 A g^{-1}	2000, (91.3%)	[7] (2015)
**RGO/NiWO4**	560 F g^{-1} at 0.8 mV s^{-1}	10000, (82 %)	[8] (2016)
**NiWO4**	1190 F g^{-1} at 0.5 A g^{-1}	2000, (90.6%)	this work
**NiWO4 //AC**	160 F g^{-1} at 0.5 A g^{-1}	5000, (92.8%)	this work

[1] Xu X, Pei L, Yang Y, Shen J, Ye M. Facile synthesis of $NiWO_4$/reduced graphene oxide nanocomposite with excellent capacitive performance for supercapacitors. Journal of Alloys and Compounds. 2016;654:23-31.
[2] Wang Y, Shen C, Niu L, Sun Z, Ruan F, Xu M, et al. High rate capability of mesoporous $NiWO_4$ –$CoWO_4$ nanocomposite as a positive material for hybrid supercapacitor. Materials Chemistry and Physics. 2016;182:394-401.
[3] Niu L, Li Z, Xu Y, Sun J, Hong W, Liu X, et al. Simple synthesis of amorphous $NiWO_4$ nanostructure and its application as a novel cathode material for asymmetric supercapacitors. ACS applied materials & interfaces. 2013;5:8044-52.
[4] Chen S, Yang G, Jia Y, Zheng H. Three-dimensional $NiCo2O_4$@$NiWO_4$ core–shell nanowire arrays for high performance supercapacitors. Journal of Materials Chemistry A. 2017;5:1028-34.
[5] He Y, Wang L, Jia D, Zhao Z, Qiu J. $NiWO_4$ /Ni/Carbon Composite Fibres for Supercapacitors with Excellent Cycling Performance. Electrochimica Acta. 2016;222:446-54.
[6] Nagaraju G, Kakarla R, Cha SM, Yu JS. Highly flexible conductive fabrics with hierarchically nanostructured amorphous nickel tungsten tetraoxide for enhanced electrochemical energy storage. Nano Research. 2015;8:3749-63.
[7] Xu X, Gao J, Huang G, Qiu H, Wang Z, Wu J, et al. Fabrication of $CoWO_4$@$NiWO_4$ nanocomposites with good supercapacitve performances. Electrochimica Acta. 2015;174:837-45.
[8] Xing X, Wang J. Reduced graphene oxide incorporated NiWO4 for high-performance energy storage. Journal of Materials Science: Materials in Electronics. 2016;27:11613-22.
